# Static and longitudinal extrauterine growth restriction in very low birth weight infants: prevalence and associated factors

**DOI:** 10.1016/j.jped.2026.101623

**Published:** 2026-09-29

**Authors:** Sudatip Kositamongkol, Claire Komarapaj

**Affiliations:** a Thammasat University, Faculty of Medicine, Department of Pediatrics, Pathum Thani, Thailand; b Thammasat University, Thammasat University Center of Excellence in Modern Technology and Advanced Manufacturing for Medical Innovation, Pathum Thani, Thailand

**Keywords:** Infant, Very low birth weight, Extrauterine growth restriction, Enteral nutrition, Neonatal nutrition, Child health

## Abstract

**Objectives:**

Extrauterine growth restriction (EUGR) occurs when preterm infants fail to achieve expected intrauterine growth rates. EUGR is classified as static (body weight < 10th percentile at 36 weeks postmenstrual age (PMA) or longitudinal (weight z-score decline > 1 standard deviation from birth to 36 weeks PMA). This study aimed to determine the prevalence of each type and identify associated factors.

**Methods:**

This retrospective cohort study included very low birth weight (VLBW) infants admitted to a tertiary-level NICU between January 2020 and December 2023. Infants who died, were transferred before 36 weeks PMA, or had chromosomal or major anomalies were excluded.

**Results:**

Of 107 infants, 55 (51.4%) had static EUGR and 35 (32.7%) had longitudinal EUGR. Multivariable analysis identified SGA as the only significant factor associated with static EUGR (aOR 9.01, 95% CI 2.08–39.08, p = 0.003). Delayed achievement of 120 mL/kg/day after day of life 10 showed a positive association with static EUGR but did not reach significance (aOR 2.57, 95% CI 0.93–7.13, p = 0.069). In contrast, delayed full enteral feeding was the only significant factor associated with longitudinal EUGR (aOR 3.98, 95% CI 1.48–10.68, p = 0.006). Agreement between definitions was weak (κ = 0.186, p = 0.019).

**Conclusion:**

Static EUGR was strongly associated with SGA status at birth, whereas longitudinal EUGR identified a partially distinct subgroup of infants. Delayed achievement of full enteral feeding was significantly associated with longitudinal EUGR. This finding warrants cautious interpretation, as delayed enteral feeding may serve as a marker of illness severity rather than a direct causal factor.

## Introduction

Advances in neonatal care have reduced mortality among very low birth weight (VLBW) infants in Thailand; however, comorbidities and suboptimal postnatal growth remain major challenges [[1], [2], [3]]. Extrauterine growth restriction (EUGR), defined as growth below the expected intrauterine trajectory, has been associated with adverse neurodevelopmental and cognitive outcomes [[4], [5], [6], [7]]. However, its definition remains controversial regarding the cut-off, timing of assessment, and classification. Static EUGR is commonly defined as body weight (BW) < 10th percentile at 36 weeks postmenstrual age (PMA), whereas longitudinal EUGR is defined as a decline in BW z-score > 1 SD from birth to 36 weeks PMA [[8], [9], [10]]. These approaches identify different groups of infants and may provide complementary information on postnatal growth. This study aimed to determine the prevalence of static and longitudinal EUGR and identify independent risk factors for each among VLBW infants.

## Methods

### Setting

This retrospective single-center cohort study was conducted from January 2020 to December 2023. The studied hospital is a tertiary care hospital in central Thailand that facilitates up to 4000 deliveries each year and is equipped with a level 3 neonatal intensive care unit (NICU) that averages 40–50 VLBW infants admitted per year. Data were retrieved from the hospital’s very low birth weight registry during 2020–2023. Parents had consented upon entry into the registry. This study was approved by the Human Research Ethics Committee of Thammasat University (Medicine) MTU-EC-PE-0–008/65.

Infants were eligible for inclusion if their birth weight was less than or equal to 1500 g and they had no multiple congenital anomalies, chromosomal abnormalities, or features suggestive of a syndromic condition. Infants were excluded if they (1) died, were referred out, or were discharged before reaching 36 weeks (PMA), or (2) had confirmed chromosomal, syndromic, or major anomalies. All VLBW infants received total parenteral nutrition within the first 6 h of life. Enteral feeds were initiated on day of life 1 per the institutional standardized feeding protocol, except where clinical judgment indicated otherwise. Feed volumes were advanced at 20–30 mL/kg/day, with most infants expected to achieve full enteral feeds of 120 mL/kg/day by day of life 10. Maternal data collected included age, gestational age, pregnancy-related comorbidities, serologic results, and mode of delivery. Infant data included birth weight, Apgar scores, need for resuscitation at birth, respiratory support, severity of respiratory distress syndrome (RDS), days to reach full enteral feeding, duration of mechanical ventilation, and morbidities such as bronchopulmonary dysplasia (BPD) and patent ductus arteriosus (PDA).

Infant body weight was measured using a Tanita digital baby scale (Model BD-815 U, Tanita Corporation, Tokyo, Japan), which provides measurements with a resolution of 10 *g* and an accuracy of ±10 *g* for weights up to 10 kg. The scale was calibrated regularly according to the manufacturer’s instructions. Body weight is routinely recorded daily in the studied unit, and the measurement obtained at 36+0 weeks postmenstrual age (PMA) was used as the assessment timepoint. Head circumference and length were not included in this study due to their more operator-dependent nature. As this was a retrospective study, measurement techniques among caregivers could not be standardized, which may have introduced imprecision.

### Definition

Static EUGR was defined as body weight 〈 10th percentile on the Fenton 2013 growth chart at 36 weeks PMA. Longitudinal EUGR was defined as a decrease in body weight z-score 〉 1 SD from birth to 36 weeks PMA. Weight z-scores were calculated using the Peditools Fenton 2013 growth calculator for preterm infants [11].

SGA was defined as birth weight < 10th percentile on the Fenton 2013 growth chart. Admission hypothermia was defined as an admission temperature < 36.5 °C. Severe RDS was defined as RDS requiring surfactant therapy.

PDA was diagnosed based on clinical signs and echocardiographic confirmation. As routine screening echocardiography was not performed, echocardiography was obtained when PDA was clinically suspected. All confirmed PDA cases were included regardless of management strategy. Infants without echocardiographic confirmation were classified as not having PDA.

During the study period, BPD was defined as supplemental oxygen requirement for > 28 days. All BPD grades were included as potential risk factors for EUGR, as fluid intake was managed cautiously even in infants with mild (grade 1) BPD.

Delayed full feeding was defined as achievement of enteral intake of 120 mL/kg/day after DOL 10. According to the standardized feeding protocol (Supplementary Material), infants without feeding delays were expected to reach 120 mL/kg/day by DOL 10. Deviations required attending physician justification and could reflect illness severity or feeding problems.

### Sample size calculation and statistical analysis

A previous study by González-García et al. reported an EUGR incidence of 72.3% [10]. This figure, together with unpublished data from Thammasat University Hospital (2014–2017) indicating an incidence of 52%, was used to calculate the required sample size. To achieve a significance level of 0.05 and a power of 80%, a minimum of 38 subjects was required. However, as this was a retrospective cohort study utilizing hospital database records, all eligible infants during the study period were enrolled.

The infants were classified into static and longitudinal EUGR groups using the 2013 Fenton growth chart and were analyzed separately for potential risk factors. Sex and postnatal comorbidities were treated as categorical variables and presented as n (%). Continuous variables were expressed as mean ± SD for normally distributed data, and as median (IQR) for non-normally distributed data.

Univariable logistic regression was performed to evaluate the crude association between each candidate variable and EUGR. Variables included in the multivariable logistic regression model were selected *a priori* based on biological plausibility and evidence from previous studies rather than on the statistical significance of the univariable analyses. Given the limited number of outcome events, the number of covariates included in the final model was restricted to reduce the risk of model overfitting. The final model included gestational age, small-for-gestational-age status at birth, delayed attainment of enteral feeding of 120 mL/kg/day, and bronchopulmonary dysplasia. Birth weight was excluded because of collinearity with gestational age. Other neonatal morbidities, including PDA, NEC, and duration of mechanical ventilation, were considered to represent overlapping aspects of illness severity or potential intermediates in the pathway between neonatal illness and postnatal growth. Therefore, these variables were not included in the primary model to minimize overadjustment, reduce redundancy among closely related clinical variables, and improve model stability given the limited number of outcome events. The objective of the multivariable analysis was to identify factors independently associated with EUGR rather than to estimate the causal effect of any single exposure. Agreement between the static and longitudinal EUGR classifications was assessed using Cohen's kappa coefficient. All statistical analyses were performed using Stata version 14 (StataCorp LLC, College Station, TX, USA).

Sensitivity analysis was performed to evaluate whether associations with EUGR were driven by baseline growth status (SGA). Specifically, all regression analyses were repeated after excluding infants who were small for gestational age (SGA) at birth, restricting the cohort to infants who were appropriate/large for gestational age (AGA/LGA) at birth. This sensitivity model used the same covariates as the primary analysis.

## Results

From a total of 148 eligible infants, 23 died, and 18 were discharged or referred out from the studied hospital before PMA 36 weeks, as shown in Figure 1. The remaining 107 infants were enrolled in the study; 55 (51.4%) had static EUGR and 35 (32.7%) had longitudinal EUGR. The mean maternal age was 30.6 ± 6.47 years, and 81 infants (75.7%) were singletons. Baseline characteristics of the infants are shown in Table 1. Of the 107 infants, 32 (29.9%) were SGA at birth; the remaining 75 non-SGA infants were included in the sensitivity analysis.

**Figure 1:**
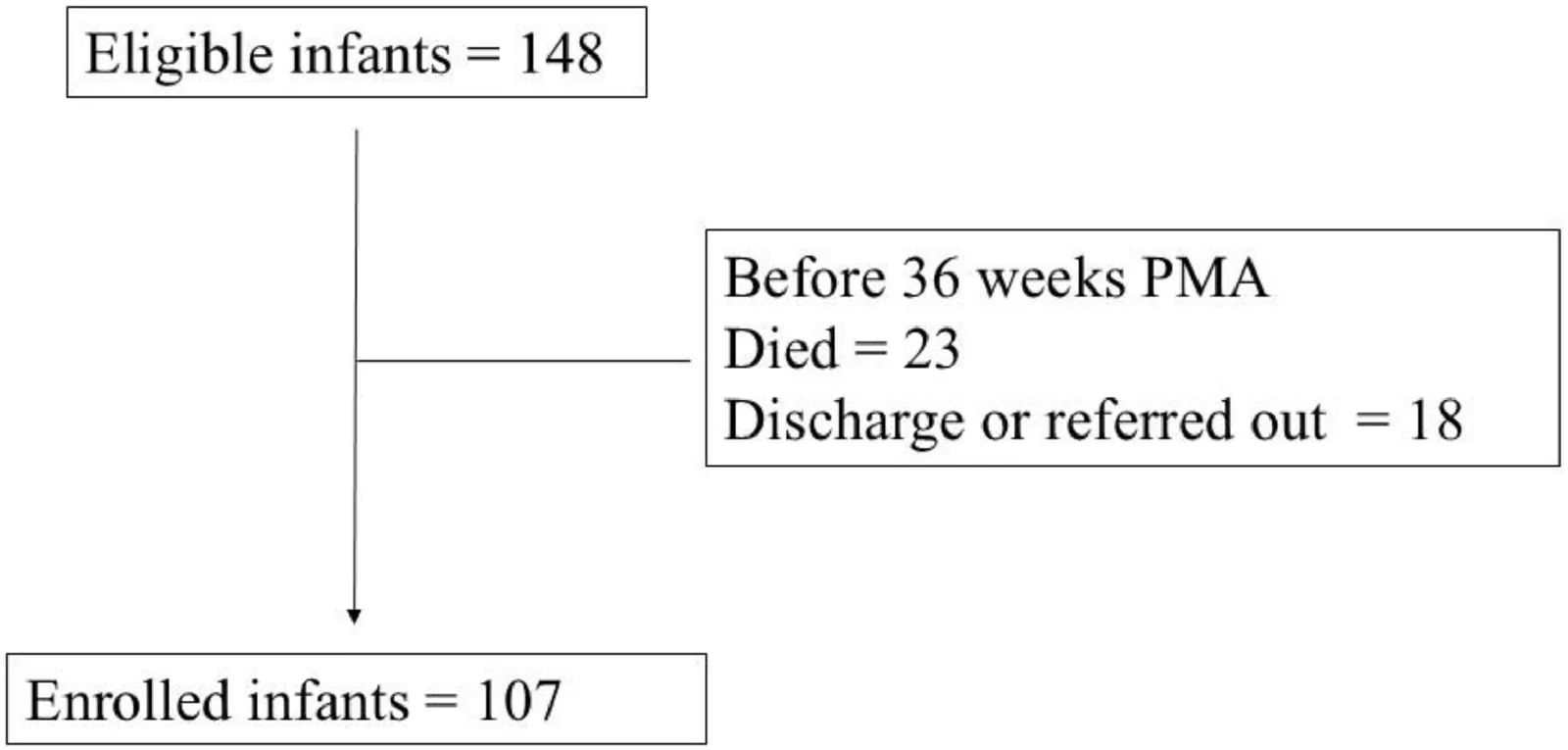
Study flow diagram.

**Table 1:** Infants’ baseline characteristics.

Continuous Variables	N = 107 median (IQR)
GA (weeks)	29.5 (28.4,32.7)
BW (g)	1180 (950,1348)
Ventilator days (day)	1 (0,15)
Categorical variables	n (%)
Male	53 (49.53)
SGA	32 (29.91)
RDS requires surfactant therapy	38 (35.5)
BPD	67 (62.6)
NEC	15 (14)
Delayed enteral feeding (failure to reach 120ml/kg by DOL10)	61 (57.01)
PDA	45 (42.45)

GA, gestational age; BW, birth weight; IQR, interquartile range; SGA, small for gestational age; RDS, respiratory distress syndrome; BPD, bronchopulmonary dysplasia; NEC, necrotizing enterocolitis; PDA, patent ductus arteriosus.

Univariate analysis showed that static EUGR was associated with higher GA and SGA at birth and maternal morbidity. RDS requiring surfactant therapy and BPD were inversely associated with static EUGR. This finding likely reflects confounding by gestational age, as infants with static EUGR had a significantly higher gestational age at birth. In the multivariable analysis, SGA at birth was the only factor independently associated with static EUGR (adjusted OR 9.01, 95% CI 2.08–39.08, p = 0.003). Delayed attainment of full enteral feeding was associated with increased odds of static EUGR, although the association did not reach statistical significance (adjusted OR 2.57, 95% CI 0.93–7.13, p = 0.069) (Table 2).

**Table 2:** Uni- and multivariable analysis of factors associated with static EUGR.

Factors	No N = 52	Yes N = 55	*p-value*	Unadjusted OR (95%CI)	*p-value*	Adjusted OR (95%CI)	*p-value*
BW	1149 (243)	1136 (247)	0.784	0.999 (0.998–1.001)	0.782		
GA	28.9 ± 2.44	31.5 ± 2.87	0.000	1.45 (1.22–1.73)	0.000	1.18 (0.88–1.59)	0.264
SGA	3 (9.4)	29 (90.6)	0.000	18.22 (5.06–65.5)	0.000	9.01 (2.08–39.08)	0.003
Male	28 (52.8)	25 (47.2)	0.442	0.94 (0.42–2.12)	0.890		
Delay full feeding	28 (45.9)	33 (54.1)	0.562	1.29 (0.60–2.77)	0.524	2.57 (0.93–7.13)	0.069
NEC	10 (66.7)	5 (33.3)	0.168	0.42 (0.13–1.33)	0.139		
PDA	26 (57.8)	19 (42.2)	0.116	0.51 (0.23–1.11)	0.089		
BPD	43 (64.2)	24 (35.8)	0.000	0.16 (0.07–0.40)	0.000	0.55 (0.12–2.46)	0.437
Maternal disease (DM,HT, Thyroid)	13 (33.3)	26 (66.7)	0.026	2.69 (1.18–6.11)	0.018		
RDS required surfactant	26 (68.4)	12 (31.6)	0.003	0.28 (0.12–0.65)	0.003		
Admission hypothermia < 36 °C	14 (43.8)	18 (56.3)	0.534	1.32 (0.57–3.04)	0.513		
Ventilator days*	3 (0,18)	1 (0,11)	0.169	0.98 (0.96–1.01)	0.194		
Breastfeeding at discharge	42 (47.2)	55 (51.4)	0.609	1.40 (0.50–3.87)	0.518		

Variables included in the multiple logistic model include: GA, SGA, Delay full feeding and BPD.

BW was excluded from multivariable analysis due to collinearity with gestational age and/or SGA status, both of which are derived in part from birth weight.

⁎ Data were presented as median (IQR), analyzed using rank-sum test. EUGR, extrauterine growth restriction; BW, birth weight; GA, gestational age; SGA, small for gestational age; NEC, necrotizing enterocolitis; PDA, patent ductus arteriosus; BPD, bronchopulmonary dysplasia; DM, diabetes mellitus; HT, hypertension; RDS, respiratory distress syndrome; OR, odds ratio; aOR, adjusted odds ratio; CI, confidence interval; IQR, interquartile range.

A sensitivity analysis excluding infants born SGA (n = 75) is presented in Table 3. After exclusion of SGA infants, no factors were independently associated with static EUGR in the multivariable analysis. Delayed attainment of full enteral feeding remained positively associated with static EUGR (OR 2.29, 95% CI 0.78–6.68, p = 0.131), although the association was not statistically significant.

**Table 3:** Sensitivity analysis of factors associated with static EUGR exclude SGA.

Factors	No N = 49	Yes N = 26	*p-value*	Unadjusted OR (95%CI)	*p-value*	Adjusted OR (95%CI)	*p-value*
BW	1157(241)	1110 (240)	0.423	1.00 (0.997–1.001)	0.423		
GA	28.7 (2.39)	29.6 (2.26)	0.147	1.17 (0.95–1.44)	0.153	1.16 (0.85–1.56)	0.346
SGA							
Male	25 (51)	11 (42.3)	0.628	0.70 (0.27–1.84)	0.473		
Delay full feeding	27 (55.1)	18 (69.2)	0.323	1.83 (0.67–5.01)	0.237	2.29 (0.78–6.68)	0.131
NEC	10 (20.4)	2 (7.7)	0.198	0.33 (0.07–1.61)	0.169		
PDA	26 (54.2)	14 (53.8)	1.000	0.99 (0.38–2.57)	0.979		
BPD	41 (83.7)	18 (69.2)	0.235	0.44 (0.14–1.35)	0.152	0.67 (0.14–3.25)	0.615
Maternal disease (DM, HT, Thyroid)	12 (24.5)	12 (46.2)	0.071	2.64 (0.96–7.25)	0.059		
RDS required surfactant	26 (53.1)	7 (26.9)	0.050	0.33 (0.12–0.92)	0.033		
Admission hypothermia < 36 °C	13 (26.5)	5 (19.2)	0.577	0.66 (0.21–2.11)	0.483		
Ventilator days*	3 (0,18)	1 (0,11)	0.679	1.00 (0.98–1.03)	0.939		
Breastfeeding at discharge	40 (81.6)	24 (92.3)	0.311	2.7 (0.54–13.56)	0.288		

Variables included in the multiple logistic model include: GA, Delay full feeding and BPD.

BW was excluded from multivariable analysis due to collinearity with gestational age and/or SGA status, both of which are derived in part from birth weight.

⁎ Data were presented as median (IQR), analyzed using rank-sum test Abbreviations: EUGR, extrauterine growth restriction; BW, birth weight; GA, gestational age; SGA, small for gestational age (omitted from multivariable model — zero variance in this subgroup); NEC, necrotizing enterocolitis; PDA, patent ductus arteriosus; BPD, bronchopulmonary dysplasia; DM, diabetes mellitus; HT, hypertension; RDS, respiratory distress syndrome; OR, odds ratio; aOR, adjusted odds ratio; CI, confidence interval; IQR, interquartile range.

Univariate analysis of the same variables showed that longitudinal EUGR was associated with lower birth weight, lower gestational age, SGA status, NEC, BPD, delayed full enteral feeding, and longer duration of mechanical ventilation. Multivariable analysis identified delayed full enteral feeding as the only statistically significant factor associated with longitudinal EUGR (aOR 3.98, 95% CI 1.48–10.68, p = 0.006) (Table 4). NEC showed a strong univariate association (OR 5.36, 95% CI 1.67–17.23, p = 0.005) but was excluded from the multivariable model due to sparse events. This association remained statistically significant after excluding SGA infants (OR 3.75, 95% CI 1.26–11.16, p = 0.018), with a magnitude of effect consistent with the primary analysis, supporting the SGA-independence of this finding (Table 5).

**Table 4:** Uni- and multivariable analysis of factors associated with longitudinal EUGR.

Factors	No EUGR N = 72	EUGR N = 35	*p-value*	Unadjusted OR (95%CI)	p-value	Adjusted OR (95%CI)	*p-value*
BW	1191 (232)	1043 (241)	0.003	0.997 (0.99–1.00)	0.004	-	-
GA	30.8 ± 3.04	29.0 ± 2.47	0.004	0.80 (0.68–0.94)	0.005	0.87 (0.66–1.14)	0.312
SGA	26 (36.1)	6 (17.1)	0.071	0.37 (0.13–1.00)	0.049	0.64 (0.18–2.28)	0.488
Male	36 (50)	17 (48.6)	1.000	0.94 (0.42–2.12)	0.890		
Delay full feeding	33 (45.8)	28 (80)	0.001	4.73 (1.83–12.21)	0.001	3.98 (1.48–10.68)	0.006
NEC	5 (6.94)	10 (28.5)	0.006	5.36 (1.67–17.23)	0.005		
PDA	25 (34.7)	20 (57.1)	0.022	2.69 (1.16–4.40)	0.021		
BPD	40 (55.5)	27 (77.1)	0.035	2.7 (1.18–6.75)	0.034	0.98 (0.23–4.25)	0.981
Maternal disease (DM, HT, Thyroid)	27 (37.5)	12 (34.3)	0.832	0.87 (0.37–2.03)	0.746		
RDS required surfactant	25 (67.3)	13 (37.1)	0.832	1.11 (0.48–2.57)	0.806		
Admission hypothermia < 36 °C	25 (67.3)	7 (20)	0.176	0.47 (0.18–1.23)	0.123		
Ventilator days*	0 (0,9)	5 (1,22)	0.001	1.04 (1.01–1.07)	0.009		
Breastfeeding at discharge	63 (87.5)	26 (74.3)	0.103	0.41 (0.15–1.16)	0.092		

Variables included in the multiple logistic model include: GA, SGA, Delay full feeding and BPD.

BW was excluded from multivariable analysis due to collinearity with gestational age and/or SGA status, both of which are derived in part from birth weight.

⁎ Data were presented as median (IQR), analyzed using rank-sum test Abbreviations: EUGR, extrauterine growth restriction; BW, birth weight; GA, gestational age; SGA, small for gestational age; NEC, necrotizing enterocolitis; PDA, patent ductus arteriosus; BPD, bronchopulmonary dysplasia; DM, diabetes mellitus; HT, hypertension; RDS, respiratory distress syndrome; OR, odds ratio; aOR, adjusted odds ratio; CI, confidence interval; IQR, interquartile range.

**Table 5:** Sensitivity analysis of factors associated with longitudinal EUGR exclude SGA.

Factors	No EUGR N = 46	EUGR N = 29	*p-value*	Unadjusted OR (95%CI)	*p-value*	Adjusted OR (95%CI)	*p-value*
BW	1182 (236)	1074 (235)	0.058	1.00 (0.996–1.000)	0.062		
GA	29.4 (2.52)	28.5 (2.02)	0.129	0.80 (0.68–0.94)	0.005	0.87 (0.65–1.16)	0.347
SGA							
Male N(%)	23 (50)	13 (44.8)	0.813	0.81 (0.32–2.06)	0.663		
Delay full feeding	22 (47.8)	23 (79.3)	0.008	4.18 (1.44–12.17)	0.009	3.75 (1.26–11.16)	0.018
NEC	4 (8.7)	8 (27.6)	0.050	4.00 (1.08–14.81)	0.038		
PDA	22 (47.8)	18 (64.3)	0.230	1.96 (0.75–5.16)	0.171		
BPD	35 (76)	24 (82.8)	0.572	1.51 (0.46–4.90)	0.494	0.76 (0.14–4.05)	0.744
Maternal disease (DM, HT, Thyroid)	13 (28.3)	11 (37.9)	0.450	1.55 (0.58–4.16)	0.383		
RDS required surfactant	21 (45.6)	12 (41.4)	0.813	0.84 (0.33–2.15)	0.717		
Admission hypothermia < 36 °C	14 (30.4)	4 (13.8)	0.164	0.37 (0.11–1.25)	0.108		
Ventilator days	3 (0,18)	9 (1,30)	0.081	1.03 (1.00–1.06)	0.043		
Breastfeeding at discharge	43 (93.5)	21 (72.4)	0.018	0.18 (0.04–0.76)	0.020		

Variables included in the multiple logistic model include: GA, Delay full feeding and BPD.

BW was excluded from multivariable analysis due to collinearity with gestational age and/or SGA status, both of which are derived in part from birth weight.

*Data were presented as median (IQR), analyzed using rank-sum test.

EUGR, extrauterine growth restriction; BW, birth weight; GA, gestational age; SGA, small for gestational age (omitted from multivariable model — zero variance in this subgroup); NEC, necrotizing enterocolitis; PDA, patent ductus arteriosus; BPD, bronchopulmonary dysplasia; DM, diabetes mellitus; HT, hypertension; RDS, respiratory distress syndrome; OR, odds ratio; aOR, adjusted odds ratio; CI, confidence interval; IQR, interquartile range.

The two definitions showed only partial overlap, with a substantial proportion of infants classified by one definition but not the other (Table 6). Of the 107 infants, 23 (21.5%) met both definitions of EUGR, 32 (29.9%) met the criteria for static EUGR only, 12 (11.2%) met the criteria for longitudinal EUGR only, and 40 (37.4%) met neither definition. Thus, only 41.8% (23/55) of infants with static EUGR also met the criteria for longitudinal EUGR, whereas 65.7% (23/35) of infants with longitudinal EUGR also met the static definition. Agreement between the two classifications was low, with an observed agreement of 58.9% and a Cohen's kappa coefficient of 0.19 (SE 0.09, p = 0.019), indicating only slight agreement beyond chance.

**Table 6:** Cross-tabulation of static and longitudinal EUGR classifications.

Static EUGR	Longitudinal EUGR No n (%)	Longitudinal EUGR Yes n (%)	Total
No	40 (37.4)	12 (11.2)	52 (48.6)
Yes	32 (29.9)	23 (21.5)	55 (51.4)
Total	72 (67.3)	35 (32.7)	107 (100)

Values are presented as n (%). Percentages are based on the total study population (N = 107).

## Discussion

Extrauterine growth restriction (EUGR) is commonly defined as a weight below the 10th percentile at either hospital discharge or between 36 and 40 weeks of gestation(8). This “static” definition yields the highest prevalence across studies [12], and may be useful for basic nutritional screening. However, static EUGR does not account for body composition, linear or head growth, nor does it adequately reflect genetic growth potential or pre-birth conditions. Most notably, its association with long-term neurodevelopmental outcomes is relatively weak when compared to longitudinal definitions [[13], [14], [15]].

Longitudinal EUGR has been defined as a decline in body weight z-score of >1 SD from birth to 36 weeks PMA. This approach incorporates birth conditions and genetic background and has demonstrated greater predictive value for neurodevelopment, including lower Bayley II Mental and Psychomotor Developmental Index scores [16,17].

In the present study, 51.4% of infants met the static criteria, and 32.7% met the longitudinal criteria. The reported prevalence of EUGR varies substantially depending on the definition and study population. For example, Yapicioglu Yildizdas et al. reported 95.3% prevalence using longitudinal criteria [18], while a study from Thailand identified 64.8% with static EUGR [19]. Agreement between the two definitions was quite weak in the studied cohort (Cohen’s kappa coefficient of 0.186), indicating that the two classifications identify largely different groups of infants and should not be used interchangeably.

Although delayed attainment of full enteral feeding was associated with more than a twofold increase in the adjusted odds of static EUGR, the confidence interval was wide and included the null value. Therefore, the present study cannot exclude either a clinically important association or no association, and larger studies are needed to obtain a more precise estimate.

The association between higher gestational age and static EUGR may appear counterintuitive but is inherent to the static definition, which classifies infants according to weight at 36 weeks' postmenstrual age regardless of their postnatal growth trajectory. Infants born at later gestational ages have less time to achieve catch-up growth before assessment at 36 weeks, whereas more immature infants have a longer interval for nutritional rehabilitation despite experiencing greater neonatal morbidity. Consequently, static EUGR may preferentially identify infants with fetal growth restriction rather than those with postnatal growth failure. The apparent inverse associations of RDS and BPD with static EUGR are likely explained by their close relationship with gestational age rather than a protective effect. More immature infants have higher rates of these morbidities but also a longer period before 36 weeks' postmenstrual age, reducing the likelihood of meeting the static EUGR definition. These findings highlight an important limitation of the static EUGR definition. Because it depends on a single anthropometric measurement at 36 weeks' postmenstrual age, static EUGR may be influenced by gestational age at birth and fetal growth status rather than reflecting true postnatal growth failure. In contrast, longitudinal EUGR, based on the decline in weight z-score over time, may better capture postnatal growth trajectory and the impact of nutritional factors.

In a sensitivity analysis restricted to infants who were not small for gestational age (SGA) at birth (n = 75), no factors were independently associated with static EUGR. Delayed attainment of full enteral feeding remained positively associated with static EUGR (OR 2.29, 95% CI 0.78–6.68), consistent with the direction of the association observed in the full cohort (adjusted OR 2.57, 95% CI 0.93–7.13), although neither association reached statistical significance.

In univariate analysis, longitudinal EUGR was associated with lower birthweight, lower GA, SGA status, NEC, BPD, delayed full enteral feeding, and longer duration of mechanical ventilation. NEC showed a strong univariable association (OR 5.36, 95% CI 1.67–17.23, p = 0.005). However, because of the small number of NEC events and the limited number of longitudinal EUGR outcomes, NEC was not included in the primary multivariable model to avoid unstable parameter estimates. In addition, NEC is closely related to illness severity and enteral feeding practices, making it difficult to disentangle its independent association within a parsimonious model.

In the multivariable model, delayed achievement of full enteral feeding was the most consistently associated factor. In the full cohort (n = 107), delayed full feeding was associated with markedly higher odds of longitudinal EUGR (aOR 3.98, 95% CI 1.48–10.68, p = 0.006), while SGA at birth was not associated with longitudinal EUGR (p > 0.05). This pattern contrasts with the static EUGR analysis (where SGA strongly predicted static EUGR) and supports the conceptual advantage of a longitudinal definition: It is less driven by birth size and more reflective of postnatal growth trajectory (i.e., growth faltering over time rather than being small at birth). Importantly, the association between delayed full feeding and longitudinal EUGR remained in the sensitivity analysis restricted to infants who were not SGA at birth (n = 75; OR 3.75, 95% CI 1.26–11.16, p = 0.018). Clinically, delayed full feeding likely captures a combination of factors—including feeding intolerance, gastrointestinal immaturity, and overall illness severity — that can limit cumulative energy/protein delivery during a critical period for growth. The present findings are consistent with previous studies demonstrating an association between inadequate nutritional intake and postnatal growth failure. Hu et al. reported that every 100-kcal/kg/day deficit was associated with a 0.1 SD decline in weight z-score [20], and Khasawneh et al. demonstrated that early caloric intake < 100 kcal/kg/day by day 15 was strongly associated with EUGR [21]. Collectively, these findings underscore the importance of careful nutritional management during the early neonatal period and suggest that infants with delayed attainment of full enteral feeding should be closely monitored for longitudinal EUGR. Breastfeeding at discharge was inversely associated with longitudinal EUGR in the non-SGA sensitivity cohort (OR 0.18, p = 0.020) but was not included in the multivariable model as it represents a discharge outcome rather than a risk factor preceding EUGR development.

The differing patterns of associated factors suggest that static EUGR is more strongly influenced by fetal growth status at birth, whereas longitudinal EUGR is more closely associated with postnatal clinical course and nutritional management. This distinction is clinically meaningful, as longitudinal EUGR may be the more appropriate measure for assessing adequacy of postnatal nutritional care. The present findings contribute to the growing evidence that nutrition remains an important and potentially modifiable factor associated with postnatal growth outcomes in VLBW infants.

This study has several limitations. First, its single-center retrospective design may limit generalizability to NICUs with different patient populations, nutritional protocols, or resources. Second, registry data lacked several potentially relevant variables, including early-onset sepsis, detailed parenteral nutrition composition, timing of breast milk fortification, and donor human milk use, resulting in potential unmeasured confounding. Third, the relatively small sample size limited the number of variables included in multivariable models, and sparse events precluded adjustment for some factors, notably NEC. Fourth, excluding infants who died, were transferred, or were discharged before 36 weeks PMA may have introduced survivor selection bias; baseline characteristics of these infants were unavailable, limiting applicability to the sickest VLBW infants. Finally, the BPD definition (supplemental oxygen > 28 gt; 28 days) included all severity grades, which may have heterogeneous effects on nutritional tolerance and growth. Severity-stratified BPD definitions may provide more precise estimates in future studies.

## Conclusion

In VLBW infants, static EUGR at 36 weeks PMA was strongly associated with SGA at birth, suggesting that it partly reflects pre-existing fetal growth restriction. Longitudinal EUGR identified a partially distinct group and was associated with delayed achievement of enteral intake of 120 mL/kg/day. These associations do not establish causality, as delayed feeding may reflect illness severity and other clinical factors. Prospective studies incorporating detailed nutritional and illness-severity data are needed to identify modifiable determinants of postnatal growth.

## Declaration of generative AI and AI-assisted technologies in the manuscript preparation process

During the preparation of this work, the author(s) used Claude (Anthropic, San Francisco, CA, USA) to assist with English language editing and manuscript preparation, including sentence refinement, grammar correction, and structural organization during manuscript revision. After using this tool, the author(s) reviewed and edited the content as needed and take full responsibility for the content of the published article.

## Funding

No funding was received for this study.

## Data availability

The de-identified datasets generated and/or analyzed during the current study are not publicly available due to patient privacy and ethical restrictions. Data are available from the corresponding author upon reasonable request, subject to approval by an appropriate institutional ethics committee.

## Conflicts of interest

The authors declare no conflicts of interest.

## Appendix Supplementary materials

Image, application 1
