# Supplementary material for: Static and longitudinal extrauterine growth restriction in very low birth weight infants: prevalence and associated factors
Source: J Pediatr (Rio J). 2026 Sep 29;103(1):101623. doi: 10.1016/j.jped.2026.101623 (PMC13654463; doi:10.1016/j.jped.2026.101623)
Supplement: Supplementary file 1 [file mmc1.docx]

**JPED-D-26-00186**

**Supplementary Material**

Standardized Feeding Regimen (Thammasat University Hospital)

Feeding Initiation

- Start minimal enteral feeding / trophic feeding at 10–20 ml/kg/day
- Initiate within the first 24 hours after birth if the infant is hemodynamically stable and does not require resuscitation
- In cases of hemodynamic instability or birth depression/asphyxia, feeding decisions are made at the discretion of the attending physician

Feeding Type

- Preferred: Human milk
  - Mother’s own milk or donor human milk
- If human milk is unavailable → use preterm formula 24 cal/oz

Advancement of Feeding

- Advance feeds according to the attending physician’s judgment or based on the feeding guideline in Table 1
- Begin checking gastric residual volume (GRV) when advancing feeds
- No need to check GRV during trophic feeding

Fortification

- Start fortified breast milk when enteral intake exceeds 80–100 ml/kg/day
- Begin with 24 cal/oz
- In infants with fluid restriction to 140 ml/kg/day, fortification may be increased to 27–30 cal/oz

Supplementation

- Iron:
  - 2–4 mg/kg/day of elemental iron, starting after 2 weeks of age
- Multivitamin:
  - At least 1 ml/day of multivitamin drops after achieving full enteral feeding
  - Administer by mixing with one feeding, due to high osmolarity

Monitoring

- Weight:
  - Measure daily
  - If weight gain is < 15–20 g/kg/day, increase caloric density to 27–30 cal/oz
- Length:
  - Measure weekly
- Laboratory monitoring:
  - ALP, calcium, phosphate, vitamin D: at 3 weeks, then every other week
  - Vitamin D levels: check as clinically indicated
  - Hematocrit, reticulocyte count, reticulocyte hemoglobin: at 3 weeks, then weekly

Feeding Advancement Table (ml × 8 feeds/day)

| BW  DOL | 500-700 | 700-900 | 1001-1250 | 1251-1500 | 1501-1750 | 1751-2000 |
| --- | --- | --- | --- | --- | --- | --- |
| 1 | 1*8 | 2*8 | 2*8 | 3*8 | 4*8 | 5*8 |
| 2 | 1*8 | 2*8 | 2*8 | 3*8 | 8*8 | 10*8 |
| 3 | 1*8 | 2*8 | 2*8 | 3*8 | 12*8 | 15*8 |
| 4 | 2*8 | 4*8 | 5*8 | 7*8 | 16*8 | 20*8 |
| 5 | 3*8 | 6*8 | 8*8 | 11*8 | 20*8 | 25*8 |
| 6 | 4*8 | 8*8 | 11*8 | 15*8 | 24*8 | 30*8 |
| 7 | 5*8 | 10*8 | 14*8 | 19*8 | 28*8 | 35*8 |
| 8 | 6*8 | 12*8 | 17*8 | 23*8 | 30*8 | 35*8 |
| 9 | 7*8 | 14*8 | 20*8 | 23-27*8 | 30-33*8 | 35-40*8 |
| 10 | 8*8 | 14*8 | 20*8 | 23-27*8 | 30-33*8 | 35-40*8 |

BW= birth weight (g), DOL= Day of life

Note: No need to check gastric content during trophic feeding.

Guideline for Infants with Gastric Residuals

Assessment

- Determine whether the infant has signs or symptoms of NEC

Warning Signs of NEC

- Abdominal distension, discoloration, or tenderness
- Systemic signs of infection (e.g. recurrent apnea, temperature instability)
- Abdominal X-ray suspicious for NEC
- Dark bilious or blood-stained gastric residuals despite correct gastric tube position
- Bloody stool

Management of gastric residual volume (GRV)

GRV < 50% of feeding volume

- Return gastric contents
- Give the next feed without subtracting the residual volume

GRV > 50% of feeding volume

- If no NEC signs:
  - Return gastric contents
  - Reduce next feeding volume by the residual amount
- If NEC signs present:
  - NPO (nothing by mouth)
  - Investigation for NEC
